# ChatGPT in Learning: Assessing Students’ Use Intentions through the Lens of Perceived Value and the Influence of AI Literacy

**DOI:** 10.3390/bs14090845

**Published:** 2024-09-19

**Authors:** Ahlam Mohammed Al-Abdullatif, Merfat Ayesh Alsubaie

**Affiliations:** Department of Curriculum and Instruction, King Faisal University (KFU), Al-Hasa P.O. Box 400, Saudi Arabia; malsebiee@kfu.edu.sa

**Keywords:** ChatGPT, AI, perceived value, AI literacy, higher education

## Abstract

This study sought to understand students’ intentions regarding the use of ChatGPT in learning from the perspective of perceived value, exploring the influence of artificial intelligent (AI) literacy. Drawing on a sample of 676 university students from diverse academic backgrounds, we employed a structured survey questionnaire to measure their perceptions of ChatGPT as a learning tool. The collected data were then analyzed using structural equation modeling (SEM) via SmartPLS 4 software. The findings showed a strong effect of the students’ perceived value of ChatGPT on their intention to use it. Our findings suggest that perceived usefulness, perceived enjoyment and perceived fees had a significant influence on students’ perceived value of ChatGPT, while perceived risk showed no effect. Moreover, the role of AI literacy emerged as pivotal in shaping these perceptions. Students with higher AI literacy demonstrated an enhanced ability to discern the value of ChatGPT. AI literacy proved to be a strong predictor of students’ perception of usefulness, enjoyment, and fees for using ChatGPT in learning. However, AI literacy did not have an impact on students’ perceptions of using ChatGPT in learning. This study underscores the growing importance of integrating AI literacy into educational curricula to optimize the reception and utilization of innovative AI tools in academic scenarios. Future interventions aiming to boost the adoption of such tools should consider incorporating AI literacy components to maximize perceived value and, subsequently, use intention.

## 1. Introduction

Advances in generative artificial intelligence (AI) have brought about a new phase of computational creativity, reshaping the limits of the content produced by machines. At their core, these technologies leverage advanced algorithms and vast datasets to generate text, images, music, and other forms of media that are remarkably similar to human-created content [1]. Generative AI technologies have found applications across various sectors, including entertainment, healthcare, and education, demonstrating their versatility and their potential to augment human capabilities in unprecedented ways [2]. Emerging from this backdrop of rapid generative AI innovation is ChatGPT, an advanced language model crafted by OpenAI in November 2022. Launched as a successor to earlier models, ChatGPT is designed to understand and generate human-like text based on the vast amount of information it has been trained on [3,4]. Since its introduction, the usage statistics for ChatGPT have been nothing short of impressive [5,6]. Millions have interacted with the model, with numerous applications developed around its capabilities, demonstrating its wide-reaching impact and transformative potential for various industries [1,5].

In the educational sector, the potential of ChatGPT is particularly promising. This technology stands out for its ability to understand and generate language-based responses, making it a valuable tool in the educational sector [7]. The model offers students and educators a unique tool for enhancing learning experiences, from facilitating personalised learning to aiding research [2,8]. For instance, its ability to generate informative responses allows students to obtain instant feedback on queries, making it an effective tool for self-study and revision [9,10,11,12,13]. Furthermore, ChatGPT provides customised learning experiences for students, assists with challenging subjects (e.g., Advanced Mathematics, Computer Programming, and Economics), and helps with assignment completion [14,15]. For educators, ChatGPT can be used as a supplementary tool to address diverse learning needs, ensuring that the content is tailored to individual student profiles [1,2,16]. Despite these benefits, the integration of ChatGPT in education also poses risks, such as fostering dependence on AI for critical thinking tasks, potential misuse for academic dishonesty, and the perpetuation of biases present in training data, as well as data privacy and security risks [17,18,19,20].

Despite the evident potential of ChatGPT in the academic realm, there is a noticeable lack of research concerning its acceptance and utilisation by students. As Rogers’ [21] diffusion of innovations theory suggests, adopting any technological tool in education is not just about its capabilities but also about its perceived utility and user acceptance. The novelty of ChatGPT, coupled with its intricate interplay of AI mechanisms, positions it distinctively in the landscape of educational technologies. However, there is a glaring gap in the literature concerning how students perceive the value of such a tool and the factors, such as AI literacy, which might affect this perception. AI literacy, which encompasses the understanding of AI principles, the ethical implications of AI, and the ability to interact effectively with AI technologies [22], plays a crucial role in shaping students’ perceptions and engagement with tools such as ChatGPT. The dynamic interplay between students’ AI literacy levels and their intention to accept and use ChatGPT in their learning emphasises the need for further investigation in this domain.

Addressing this gap is paramount, especially as educational institutions worldwide are at the cusp of a technological revolution, with AI-driven tools, such as ChatGPT, poised to play a pivotal role. This study aims to explore students’ intentions to use ChatGPT, shedding light on the perceived value of the tool and the influencing role of AI literacy in shaping these perceptions. Understanding the factors that influence students’ acceptance and use of ChatGPT in learning can guide the development of more effective educational policies and practices that foster the successful adoption of such tools. This study can contribute to the broader discourse on the role of AI literacy in shaping students’ perceptions of the value of using ChatGPT. The value-based model (VAM) by Kim et al. [23] guides this investigation among Saudi students in higher education. This study delves into the alternative perspective—how AI literacy contributes to our understanding of ChatGPT adoption behaviour.

## 2. Theoretical Foundation

### 2.1. Value-Based Model (VAM)

The existing body of literature includes a variety of theories and models that examine individuals’ perceptions of embracing and integrating new technologies. The VAM represents a conceptual framework that plays a pivotal role in understanding the dynamics of technology adoption within various contexts [23]. Rooted in the seminal work of Venkatesh and Davis [24], the VAM extends and refines the technology acceptance model (TAM) [25], incorporating the influence of perceived value, as defined by Zeithaml [26], on user acceptance and adoption of technology. It emphasises the significance of perceived value and is an influential indicator of intention to use and willingness to accept [27]. According to Kim et al. [23], the core of the VAM lies in the notion of perceived value, emphasising users’ subjective assessments of the benefits and costs associated with adopting a particular technology. In terms of benefits, perceived value encompasses both utilitarian aspects, relating to the system’s functionality and efficiency, and hedonic aspects, encompassing the system’s capacity to evoke enjoyment or pleasure during use. In terms of costs, perceived fees and perceived risks represent crucial elements that users evaluate when considering the adoption of a new technology. These factors contribute significantly to users’ perceptions of value and can shape their decisions to accept or reject a particular technology. Kim et al. [23] proposed that, when assessing the intention to use technology, perceived value is determined by two main factors: the benefits individuals derive (such as perceived usefulness and perceived enjoyment) and the relative sacrifices they make (including perceived fees and perceived risk). Within the context of this study, perceived value reflects how students assess the balance between the advantages and disadvantages of using ChatGPT. Students who perceive ChatGPT as a valuable tool for enriching their learning experiences are more likely to accept and integrate it into their educational practices.

#### 2.1.1. Perceived Benefits and Perceived Value

The VAM revolves around perceived benefits, a pivotal determinant shaping users’ technology acceptance. Perceived usefulness, according to the VAM, is one of the components of perceived benefits. In this study, we conceptualise perceived usefulness as students’ belief in ChatGPT’s potential to enhance their learning performance. This enhancement could manifest in several ways, such as deeper comprehension of concepts, enhanced interaction with educators, or support for involvement with educational content. These enhancements may lead students to perceive the advantages of integrating ChatGPT into their learning as outweighing any associated drawbacks. According to Yu et al. [28], the most significant factor affecting the adoption of media tablets is perceived usefulness, particularly when moderated by perceived value. Likewise, Liao et al. [29] confirmed that, in adopting an e-learning system, perceived value is strongly predicted by perceived usefulness. Comparable findings have been reported in other studies [30,31,32]. Consequently, we posit the first hypothesis as follows:

**Hypothesis** **(H1):** *Perceived usefulness positively influences the perceived value of ChatGPT.*

While perceived usefulness plays a crucial role in providing functional advantages, perceived enjoyment is equally vital for its emotional benefits, as outlined by Teo [33]. In line with the VAM, the influence of perceived enjoyment on perceived value is emphasised in predicting technology adoption [23]. Beyond learning improvements, students derive exciting and enjoyable benefits from interacting with ChatGPT. Perceived enjoyment indicates the level of engagement, prompt feedback, and user-friendly design, the latter being one of the main reasons ChatGPT models became popular in 2022 despite having been around since 2018 [34]. Consequently, in this study, perceived enjoyment denotes how much students perceive ChatGPT usage as offering engaging and enjoyable learning experiences. By providing a gratifying and pleasurable learning atmosphere, ChatGPT can inspire students to participate more actively, resulting in enhanced learning results. Numerous studies have highlighted a strong and significant relationship between perceived enjoyment and perceived value in adopting technologies [29,31]. In the AI-driven context, Sohn and Kwon [35] highlighted that perceived enjoyment emerged as the foremost predictor of users’ perceived value in utilising AI-based intelligent products. Likewise, a recent investigation conducted by Al-Abdullatif [17] concerning chatbot acceptance among university students demonstrated that perceived enjoyment stood out as the primary predictor of perceived value. Thus, the second hypothesis is proposed as follows:

**Hypothesis** **(H2):** *Perceived enjoyment positively influences the perceived value of ChatGPT.*

#### 2.1.2. Perceived Sacrifice and Perceived Value

The VAM places significance on perceived sacrifice as a crucial factor influencing adoption decisions. This involves considering both monetary and non-monetary aspects [36]. The non-monetary aspect encompasses intangible risks related to privacy and accuracy concerns and the fear of overreliance on the tool. While OpenAI has stated that ChatGPT does not store confidential information or use it to train its models, the company runs closed-source software, making verification of such claims impossible [34]. When using ChatGPT, students must consider the accuracy and reliability of the responses generated by ChatGPT: security, privacy, and ethical concerns [1,37,38,39,40,41]. If there is a risk of misinformation or of the system providing inaccurate responses, students might hesitate to rely fully on ChatGPT for important tasks or information. In addition, students may worry about the confidentiality of the information they share with ChatGPT, especially if the system retains conversations or if there is potential for data breaches. Concerns about biases in responses, inappropriate content generation, or academic integrity may influence students’ willingness to accept and adopt ChatGPT. This study concluded that students’ intentions to use ChatGPT were negatively influenced by these risks, outweighing the potential benefits [42]. A growing body of research supports the notion that an inverse correlation exists between perceived risks and perceived value when predicting technology use intention [28,29,30,36,43], leading to the formulation of the third hypothesis:

**Hypothesis** **(H3):** *Perceived risk negatively influences the perceived value of ChatGPT.*

The relationship between perceived fees and perceived value plays a pivotal role in understanding students’ intentions to use ChatGPT. Perceived fees, within the monetary context of the VAM, encompass the financial costs associated with adopting and utilising new technology [23]. This extends beyond initial acquisition or licensing charges, encompassing ongoing expenses, such as upgrades and additional costs tied to technology utilisation. In the context of ChatGPT, the perceived fees are closely tied to the tangible financial considerations and risks associated with subscribing to the service. While ChatGPT-3.5 is available free of charge, the more advanced ChatGPT-4 requires a subscription from users. Students, as potential users, engage in a delicate balance between these perceived fees and the benefits offered by ChatGPT [44]. Research indicates that the degree of perceived fees has a notable influence on customers’ perceived value [29,43,45,46,47]. This is true in the context of ChatGPT, where students evaluate the financial implications against the perceived benefits. If the benefits derived from the service exceed the associated costs, students are more likely to develop an intention to use ChatGPT.

Conversely, if the perceived value falls short compared to the perceived fees, students may decline or reconsider the adoption of the service. Students engaging with ChatGPT may express concerns about subscription fees or the costs associated with its use. The decision-making process involves carefully assessing whether the benefits derived from ChatGPT, such as enhanced learning or productivity, justify the financial investment required. This complex interplay between perceived fees and perceived value becomes a critical determinant in shaping students’ intentions to use ChatGPT. Therefore, the following hypothesis was derived:

**Hypothesis** **(H4):** *Perceived fees negatively influence the perceived value of ChatGPT.*

#### 2.1.3. Perceived Value and ChatGPT Use Intention

In consumer behaviour research, it is well established that consumers’ attitudes toward a product are greatly influenced by its perceived value [48]. Perceived value, according to Zeithaml [26], refers to the “overall assessment by consumers of the utility of a product based on perceptions of what is received and what is given” (p. 14). In this study’s context, it is expected that the perceived value of ChatGPT will increase as students’ learning experiences become enriched with more benefits and fewer associated risks. The perceived value of ChatGPT lies in its ability to create personalised learning environments and enhance students’ academic performance [39,49,50,51,52]. In the field of information system research, perceived value is commonly acknowledged as a robust predictor of technology acceptance [17,28,31,32,53,54]. In terms of ChatGPT perceived value, the following hypothesis is proposed:

**Hypothesis** **(H5):** *Perceived value positively influences the acceptance of ChatGPT.*

### 2.2. The Role of AI Literacy

AI literacy is a newly emerging concept that seeks to define, teach, and evaluate the knowledge and skills related to AI technology [22]. AI literacy refers to individuals’ ability to comprehend, utilise, monitor and critically evaluate AI applications, even without the ability to develop AI models themselves [22,52,55,56]. Long and Magerko [57] provided a widely referenced definition of AI literacy: “a set of competencies that enables individuals to critically evaluate AI technologies, communicate and collaborate effectively with AI, and use AI as a tool online, at home, and in the workplace” (p. 2). This comprehensive approach to AI literacy aims to empower individuals, enabling them to navigate and contribute to an increasingly AI-driven world.

The increasing incorporation of AI into teaching and learning environments has sparked interest in AI literacy [58]. Students must learn to use AI technologies wisely and discern between ethical and unethical practices [41,59]. An individual’s literacy skills are important in conducting learning activities [60,61]. Several researchers have highlighted the positive effects of high AI literacy on human–AI interactions. For example, Su and Yang [62] noted improvements in students’ AI knowledge, skills, and attitudes from AI literacy programmes, indicating that AI literacy can positively influence learners’ perceptions and abilities. Wang et al. [58] indicated that the level of AI literacy determines whether students embrace AI technologies.

#### 2.2.1. AI Literacy and Perceived Benefits

Higher AI literacy enables users to better understand the capabilities and limitations of AI technologies, leading to more effective adoption and usage [22,58]. Users with a strong grasp of AI can better decide which AI tools and solutions are most beneficial for their specific needs [56]. They can critically evaluate different AI offerings, leading to a more nuanced understanding of the benefits these technologies can provide. Lee et al. [63] explored the effect of information literacy on users’ trust in using government websites. They found a positive effect of users’ perceived information literacy on users’ perceived usefulness. Likewise, the relationship between AI literacy and the perceived enjoyment of adopting AI technology is significant. As users become more literate in AI, their understanding, skills, and attitudes towards these technologies improve, increasing their enjoyment and willingness to adopt AI. This is particularly evident in educational settings, where both students’ and teachers’ perceptions of the usefulness and enjoyment of AI influence their adoption and use of these technologies [17]. Feng [64] explored the effect of digital literacy in predicting online learning self-efficacy among Chinese students in second language learning. The findings revealed that digital literacy could predict about 95% of the changes in the students’ perceived enjoyment. The results suggest that digital literacy strongly influences perceived usefulness. Therefore, the following hypotheses are proposed:

**Hypothesis** **(H6):** *AI literacy positively influences the perceived usefulness of ChatGPT.*

**Hypothesis** **(H7):** *AI literacy positively influences the perceived enjoyment of ChatGPT.*

#### 2.2.2. AI Literacy and Perceived Sacrifice

The rise of AI models, especially conversational models such as ChatGPT, in the academic context has brought both advantages and potential challenges [65]. For many users, the sacrifice of using such technologies, whether in terms of accuracy and reliability, privacy, misconduct, fee risks, or other metrics, is a significant consideration. The degree to which users perceive these risks may be influenced by their understanding and knowledge of AI—a concept we refer to as AI literacy. Therefore, understanding how AI literacy relates to risk perception is paramount. Users’ AI literacy significantly impacts their perception of the risks associated with AI technology [66]. Higher AI literacy might correlate with a more nuanced understanding of AI’s benefits and limitations, potentially leading to more informed and less apprehensive attitudes towards AI adoption. Conversely, lower AI literacy could result in heightened apprehension due to misunderstandings or a lack of information about AI’s capabilities regarding risks and fees. Bender et al. [67] indicated that students’ and faculty’s understanding of the risks and impacts of AI models is central to AI literacy.

Fütterer et al. [68], in their article on global reactions to AI innovations, suggested that AI, such as ChatGPT, stressed the importance of AI literacy in shaping perceptions of the risks and opportunities associated with ChatGPT in education. Regarding users’ perceived fees, the findings of a recent systematic literature review by Casal-Otero et al. [69] highlighted how AI literacy can significantly impact users’ awareness of AI technology costs. Their findings suggest that a user’s Al literacy level can significantly affect their awareness and knowledge of the costs associated with AI technology purchases and usage. When students are more knowledgeable about ChatGPT, they can better understand the cost factors involved, such as purchasing, upgrading and potential use training. In this study, AI literacy correlates with a more nuanced understanding of ChatGPT’s capabilities and limitations, influencing how users perceive and mitigate its associated risks and fees. Therefore, the following hypotheses are proposed:

**Hypothesis** **(H8):** *AI literacy negatively influences the perceived risk of ChatGPT.*

**Hypothesis** **(H9):** *AI literacy positively influences the perceived fees relating to ChatGPT.*

#### 2.2.3. AI Literacy and Perceived Value

In the digital literacy context, skilled users are more likely to explore advanced features and use technology more effectively, leading to richer and more satisfying experiences [70]. A study by Vekiri [71] indicated that digital literacy not only affects the ability to use technology, but also influences the affective and motivational aspects of technology use. Hargittai and Hsieh [72] found that higher levels of digital literacy are associated with more positive attitudes towards the internet and its capabilities, suggesting that those with greater digital literacy are more likely to value using online technologies. Several other studies have indicated the positive impact of digital and information literacy on technology adoption [60,73,74]. Regarding AI literacy, Wang et al. [58] affirmed a notable correlation between AI literacy and attitudes toward robots’ and users’ regular use of AI. Luckin and Holmes [75] demonstrated that teachers with higher AI literacy were more likely to value AI tools in the classroom because of their potential to personalise learning and improve student outcomes.

Therefore, users with higher AI literacy are better equipped to leverage the full potential of AI technologies, leading to a more efficient and effective user experience [52,58]. This enhanced experience directly correlates with a higher perceived value of the technology. With adequate AI literacy, users can make more informed decisions regarding the adoption and use of AI technologies [55]. They are more capable of evaluating the suitability, risks and benefits of AI solutions, enhancing the perceived value of these technologies [22]. AI literacy helps users understand AI’s ethical and social implications, influencing how they value its integration into specific contexts. In this study, students who were literate in AI technologies perceived greater value in ChatGPT due to its potential to enhance their learning experiences, leading to more use intention. This relationship underlines the importance of promoting AI literacy to facilitate the successful and satisfying adoption of ChatGPT. With these considerations, the following hypothesis is proposed:

**Hypothesis** **(H10):** *AI literacy positively influences the perceived value of ChatGPT.*

## 3. Method

### 3.1. Data Collection and Participants

In November 2023, a survey was conducted among university students at both undergraduate and postgraduate levels after securing ethical clearance and informed consent as per the university’s ethics committee guidelines (details omitted for blind review). The survey was disseminated through university email systems and various social media channels. Participants were allowed a four-week period to voluntarily respond to the survey. The sample comprised 83.9% females, 91.6% undergraduates, and 86.6% within the age range of 19 to 22, representing various academic disciplines as detailed in Table 1.

### 3.2. Data Analysis and Measurement

**Figure 1:**
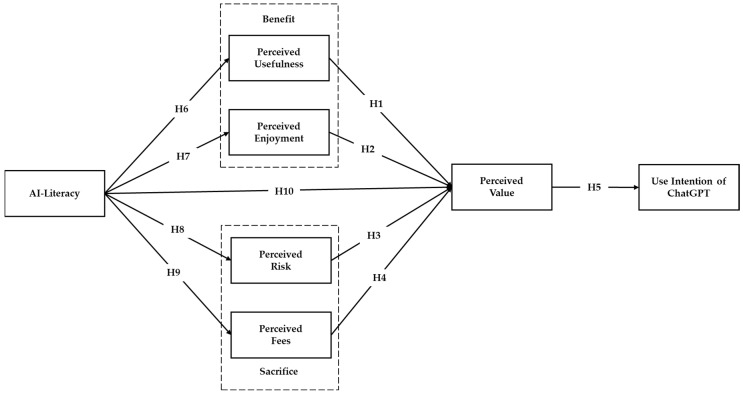
The proposed research model.

Data were analyzed using the partial least squares structural equation modeling (PLS–SEM) approach through SmartPLS 4.0 software. PLS–SEM is particularly suited for exploratory research where the goal is to assess complex relationships and develop theoretical insights. Given that the study explores the relationships of the factors influencing students’ intentions to use ChatGPT, PLS–SEM provides a robust framework for examining these intricate relationships. According to Hair et al. [76], PLS–SEM involves two critical phases: assessment of the measurement model followed by evaluation of the structural model to validate 10 research hypotheses concerning the constructs. The evaluation of participants’ perceptions concerning the 7 constructs of the research model (see Figure 1) utilized a pre-established survey instrument comprising 32 items. To ensure the precision and suitability of the survey items, three educational technology experts reviewed and slightly modified the wording. Data collection began with demographic information, followed by assessment of perceptions using a 5-point Likert scale. The constructs assessed include perceived usefulness (3 items), perceived enjoyment (3 items), perceived fees (3 items), and perceived value (4 items), adapted from Kim et al. [23] and Liao et al. [29]; perceived risk (5 items), adopted from Kim et al. [23] and Sallam et al. [77]; use intention (4 items), adapted from Liao et al. [29] and Marjerison et al. [40]; and AI literacy (10 items), adapted from Wang et al. [58] and Zhao et al. [52].

## 4. Results

### 4.1. Measurement Model Analysis

The process began with the validation of construct validity to ensure accurate representation of the intended concepts [76]. Table 2 illustrates the indicator loadings, with all values between 0.72 and 0.93 indicating satisfactory to high levels of loading, as recommended by Hair et al. [76,78]. Internal consistency was confirmed through Cronbach’s alpha (α) and composite reliability (CR), with values (0.80 ≥ α ≤ 0.93, 0.80 ≥ CR ≤ 0.97), indicating good to high reliability. As advised by Hair et al. [76,78], convergent validity was affirmed through the average variance extracted (AVE) for each construct (AVE > 0.5). 

Discriminant validity was established through the heterotrait–monotrait ratio (HTMT), ensuring distinctiveness among constructs. Ideally, HTMT < 0.85 indicates valid discriminant validity. Table 3 illustrates that the square roots of the AVE values for all constructs exceed their respective intercorrelations, with HTMT values consistently below 0.85. 

### 4.2. Structural Model Analysis

**Figure 2:**
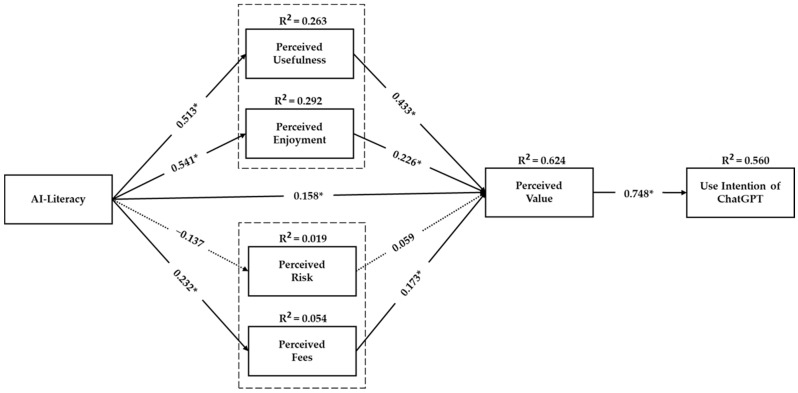
Standardized path coefficient results (* *p*-value < 0.001).

The focus then shifted to the structural model, where the relationship between constructs was analyzed. To this end, Hair et al. [76,78] suggested the presentation of values, such as standardized path coefficients (β), standard errors (SE), t-values (t), and (*p*-values). The results, summarized in Table 4 and Figure 2, demonstrated significant direct effects of several constructs on perceived value and user intention, confirming multiple hypotheses; perceived usefulness, perceived enjoyment, perceived fees, and AI literacy had direct significant effects on students’ perceived value of using ChatGPT in learning, confirming H1, H2, H4, and H10. However, perceived risk showed no significant effect on perceived value, rejecting H3. Furthermore, the results indicate that AI literacy positively influences students’ perceived usefulness, perceived enjoyment, and perceived fees, confirming H6, H7, and H9. However, the results revealed that perceived risk was not predicted by AI literacy, causing the rejection of H8. In addition, it was found that use intention was positively predicted by perceived value, which supports H5. The R^2^ values indicated a high level of predictive capability for key outcomes, with Q^2^ values supporting the model’s predictive relevance for out-of-sample validation [78,79]. This suggests that the model’s ability to anticipate students’ acceptance of ChatGPT integration in learning contexts (R^2^ and Q^2^ values are shown in Table 3).

## 5. Discussion and Implications

Like all technological innovations, the efficacy and impact of ChatGPT in the educational sector hinge not just on its capabilities, but also on its acceptance by end users—students, educators, and academic institutions. This is where the extant literature falls short. While there is abundant discourse on the potential of AI in education or even the theoretical underpinnings of AI-driven tools, empirical investigations specifically into ChatGPT’s acceptance in academic settings are limited. Exploring the interplay among perceived value, AI literacy, and students’ intentions to use ChatGPT is crucial for realising its full educational potential. To this end, this study seeks to investigate university students’ intentions to use ChatGPT, using critical factors of the VAM and the influencing role of AI literacy in shaping these perceptions. This research sets itself apart from existing studies by incorporating factors associated with the VAM—such as perceived usefulness, perceived enjoyment, perceived risk, perceived fees, and overall perceived value—along with AI literacy to forecast student intentions to use ChatGPT for educational purposes. It enriches the current knowledge base about ChatGPT. Furthermore, it offers practical recommendations for stakeholders in the education sector, including policymakers, educators and students, to facilitate its effective integration into Saudi higher education. This section reviews the research findings and their broader implications.

### 5.1. Perceived Usefulness and Perceived Value

The support for H1, which posits that perceived usefulness positively influences the perceived value of ChatGPT, provides deep insights into the adoption and continued use of technological innovations in educational settings. This relationship underscores a fundamental aspect of TAMs, such as the VAM [23], where the usefulness of a tool significantly impacts its perceived value and, consequently, its adoption rate among users. This aligns with the findings of numerous prior research studies [28,29,30,31,32]. This finding suggests that university students, in particular, value ChatGPT for its utility in completing academic tasks, such as writing assistance, research, and learning new concepts [1,7,20,65]. This utility makes the tool more attractive and embeds it as a critical resource in the students’ educational toolkits. The positive influence of perceived usefulness on the tool’s value indicates that students are pragmatic in their technology adoption choices. Tools that directly contribute to their academic success or make their study processes more efficient are more likely to be valued and used.

Given the strong influence of perceived usefulness on the perceived value of ChatGPT, educational policymakers should prioritise the seamless integration of AI tools into academic programmes where they directly enhance learning outcomes. Policymakers could advocate for institutional investments in AI technologies tailored to students’ academic needs, ensuring that ChatGPT and similar tools are embedded across a wide range of subjects. Curriculum designers can amplify the perceived value of ChatGPT by aligning its features with key academic tasks, such as research, writing and critical thinking exercises, making the tool indispensable for academic success. In practice, this could involve designing specific assignments or projects that require the use of AI tools to complete, reinforcing their utility in real-world applications. Additionally, offering targeted workshops and tutorials on maximising the use of AI for academic purposes can help students better understand how to leverage the technology to improve performance. Institutions should also consider creating more intuitive interfaces and integrating AI tools with other learning platforms, making ChatGPT more accessible and user friendly for a broader range of students.

### 5.2. Perceived Enjoyment and Perceived Value

The confirmation of H2, which states that perceived enjoyment positively influences the perceived value of ChatGPT, sheds light on the emotional and experiential aspects of technology adoption among university students [23]. This relationship emphasises that, beyond practical value, the enjoyment derived from interacting with ChatGPT significantly contributes to its overall value to users. The enjoyment factor is crucial in keeping users engaged and motivated to use ChatGPT [17,29]. Numerous studies have emphasised the significant and favourable connection between perceived enjoyment and perceived value in adopting technology [29,31]. In AI-based technology environments, similar findings have been reported in numerous studies [17,35]. If students find pleasure in interacting with ChatGPT, whether through its conversational style, the novelty of AI interaction, or the discovery of new information, they are more likely to use it frequently and explore its capabilities further. This finding underscores the importance of a positive user experience, where ease of use, interface design and interactive feedback play critical roles.

This result implies that policymakers should focus on fostering an academic environment that values utility and the engagement and satisfaction students derive from using technology. This can be done by promoting AI tools in creative and interactive learning scenarios, such as through gamified learning environments where students can enjoy interacting with ChatGPT while achieving educational goals. To enhance learning outcomes and enjoyment, curriculum designers should consider embedding ChatGPT in collaborative activities, such as group discussions, brainstorming sessions and problem-solving challenges. Highlighting the novel and engaging aspects of AI interaction, such as personalised feedback, conversational learning, and self-paced exploration, can make ChatGPT academically beneficial and enjoyable. Incorporating user-friendly designs that prioritise ease of use, personalisation, and interactive features would enhance student engagement.

### 5.3. Perceived Risk and Perceived Value

The VAM suggests that technologies perceived as having lower risks are more likely to be valued and adopted by users [36]. In utilising ChatGPT, students should consider aspects such as the accuracy and dependability of ChatGPT’s generated responses, alongside concerns related to security, privacy, and ethics [1,37,38,39,40,41]. Numerous studies have endorsed the concept that perceived risks and perceived value share a reverse correlation when it comes to predicting the acceptance of technology [28,29,30,36,43]. The result for H3, which posited that perceived risk negatively influences the perceived value of ChatGPT but was not supported, presents a nuanced understanding of how users perceive and value AI technologies, such as ChatGPT, despite potential risks. Students, particularly those in higher education, are increasingly exposed to various forms of technology. This exposure may have led to a normalisation of risks associated with technological tools, making students less sensitive to the risks when they perceive high utility and enjoyment. In the case of ChatGPT, students may prioritise the tool’s academic utility and efficiency in assisting with tasks such as research, writing, and concept clarification, effectively downplaying concerns about data privacy or accuracy. This reflects a cost–benefit trade-off where the perceived value of the tool outweighs the potential risks.

The role of experience and engagement might be another justification for this result. Students who have more experience engaging with AI tools are better at navigating risks and understanding the limitations of these technologies. This familiarity reduces their concern for potential issues, such as bias in the AI’s responses or data privacy, making risk perception less salient. Furthermore, the immediacy of benefits (e.g., instant responses, enhanced learning) may make the risks appear distant or abstract, particularly when students do not encounter significant negative experiences while using ChatGPT. As ChatGPT and similar AI tools become more prevalent, students may also feel that awareness and transparency around risks have improved, leading to the assumption that any remaining risks are manageable or acceptable. For instance, privacy policies, usage guidelines and ethical considerations provided by universities or AI developers might alleviate concerns, further contributing to the diminished perception of risk in relation to value.

This finding has significant implications for how AI tools are presented and integrated into the educational environment. For example, universities should highlight the immediate and practical benefits of using ChatGPT to ensure that students understand how it can directly support their learning processes. At the same time, institutions should promote transparency regarding the risks of AI tools by providing clear privacy policies, usage guidelines, and ethical considerations. However, given the reduced sensitivity to risk, these efforts can be framed in a way that reinforces students’ existing trust in the tool and focuses on practical strategies to enhance engagement and positive experiences. While perceived risk may not heavily influence value, institutions are still responsible for addressing and communicating potential risks clearly. By ensuring transparency and ethical usage guidelines, they can continue to build trust without causing students to fixate on perceived dangers. Universities can also maintain institutional safeguards and best practices to ensure the responsible use of AI tools, ensuring that low-risk perception remains warranted. Institutions and developers should continuously monitor users’ perceptions and experiences of ChatGPT, including any concerns about risks that might arise as the technology evolves. Encouraging and promoting the ethical use of ChatGPT and similar tools can further reduce perceived risks. Guidelines, best practices and examples of ethical use can help students navigate how to use AI technologies responsibly, enhancing their overall value.

### 5.4. Perceived Fees and Perceived Value

The link between perceived fees and perceived value is crucial in understanding users’ willingness to use technology. Within the framework of the VAM, perceived fees refer to financial expenses related to adopting and using new technologies [23]. In using ChatGPT for learning, this cost can significantly influence students’ perceived value of the technology. The findings of this study confirmed this result H4. Studies have shown that the level of perceived fees significantly influences users’ perceived value of a service or product [29,43,45,46,47]. The support for H4 suggests that students are sensitive to the costs associated with using ChatGPT and that this sensitivity can detract from the tool’s perceived value. This is important in educational contexts, where budgets are often limited, and students may have to weigh the benefits of paid tools against other necessary expenses. The negative impact of perceived fees on ChatGPT’s value indicates that, as the cost of using the tool increases, its attractiveness decreases unless the fees are justified by exceptional benefits or outcomes that cannot be obtained elsewhere for free or at a lower cost.

Educational policymakers might explore options to reduce financial barriers by negotiating institutional licences or bulk pricing with AI providers, ensuring that students can access these tools without prohibitive costs. This is especially important for students from lower socio-economic backgrounds who may not have the resources to pay for premium AI features. Institutions could also consider offering tiered access models where basic features are free or subsidised, with more advanced functionalities available at a reduced rate for students. Curriculum designers can collaborate with AI providers to create free or discounted packages that align with specific academic programmes, ensuring that students can access the features most relevant to their studies. Additionally, transparent communication about the costs involved in AI development and the value these tools bring to the academic experience can help students better appreciate the reasons behind pricing models. Institutions should also implement feedback mechanisms to gather students’ opinions on pricing strategies and perceived value, using this input to inform future partnerships and pricing adjustments.

### 5.5. Perceived Value and ChatGPT Use Intention

The support for H5, which states that the perceived value of ChatGPT positively influences students’ intention to use it, is a pivotal finding with significant implications for adopting and utilising technological tools in educational settings. This outcome supports previous findings on the acceptance of chatbots [17,80,81,82]. This relationship underscores the integral role of perceived value in determining how likely students are to incorporate ChatGPT into their academic practices. This finding indicates that students’ intentions to use ChatGPT are heavily influenced by their overall assessment of its value, encompassing usefulness, enjoyment, cost, and other factors. A tool that offers significant benefits, whether through enhancing academic performance, providing enjoyment or being cost-effective, is more likely to be adopted and regularly used [36]. Students consider isolated features or capabilities and evaluate ChatGPT as a whole. This holistic assessment means that improvements in any aspect that increases perceived value can positively impact students’ intentions to use the tool.

For policymakers, enhancing the intrinsic value of ChatGPT involves not just highlighting its utility but fostering an environment where its application is seen as indispensable. This can be achieved by focusing on targeted innovations that leverage the platform’s strengths, such as providing tailored solutions for personalised learning, supporting various learning styles, and automating administrative tasks, like grading or feedback. For academic institutions, integrating ChatGPT into existing academic ecosystems is critical for encouraging its widespread use. Embedding it into learning management systems, like Blackboard or Moodle, allows seamless access for students and educators, making ChatGPT a natural extension of the tools they already use. For example, universities could introduce ChatGPT-powered features such as on-demand tutoring, automated study guides, or interactive feedback within assignments, thus making it a routine part of the student experience. This increases the frequency of use and normalises the tool’s presence in daily academic workflows.

Moreover, making ChatGPT available through library resources or within course syllabi as a recommended tool for research and writing can enhance its perceived credibility. Institutions might also host collaborative projects in which students and educators use ChatGPT to tackle complex subjects, reinforcing its potential as a cooperative learning aid rather than a passive tool. Finally, providing workshops, tutorials and hands-on training sessions can significantly enhance the perceived value of ChatGPT among students. Offering such resources ensures that students are aware of the tool and confident in using it effectively to improve their academic work. Faculty members could similarly be trained on the advanced functionalities of ChatGPT, allowing them to more effectively guide students on its best uses. These initiatives also reduce the learning curve, fostering greater adoption and maximising the tool’s impact on learning outcomes.

### 5.6. AI Literacy and Perceived Usefulness

The finding that H6 is supported reveals that AI literacy, or the degree to which individuals understand and are familiar with AI concepts and technologies, positively impacts how useful they perceive ChatGPT to be. This relationship underscores the importance of knowledge and education in shaping the perceptions and adoption of innovative technologies in educational settings. Students with a higher level of digital and information literacy will likely have a deeper understanding of technologies’ capabilities and limitations [63]. Their ability to leverage the tool efficiently contributes to their perception of the AI tool as highly useful [22,58,62]. With a solid understanding of ChatGPT, students might be more open to integrating it into their study routines, recognising its potential to aid research, writing and learning. Knowledgeable users are better equipped to set realistic expectations for what ChatGPT can offer. This alignment between expectations and capabilities can lead to higher satisfaction with the tool’s performance, further enhancing its perceived usefulness.

AI literacy programmes should be designed to go beyond basic knowledge. These programmes can introduce students to the underlying mechanisms of AI, such as machine learning, natural language processing and the ethical considerations of AI in education. A deeper understanding of how ChatGPT operates can demystify the tool and encourage students to see it as a powerful resource, rather than just a black-box technology. When students understand the processes that make ChatGPT function—such as how it generates answers based on large datasets—they are more likely to trust its outputs and use it strategically. Moreover, structured AI literacy programmes could focus on specific applications of AI in academic work. Tutorials, workshops, or courses might cover how AI can assist in research, problem solving, or content creation, guiding students on how to integrate ChatGPT into various stages of their academic processes. For example, showing students how ChatGPT can assist with brainstorming ideas, generating outlines or summarising large bodies of text can clarify its usefulness, likely increasing student engagement with the tool.

Furthermore, embedding AI literacy components into existing curricula allows AI education to be part of a broader learning strategy, ensuring that students develop technical skills and a critical understanding of how to use AI responsibly and effectively. For example, AI literacy could be introduced in introductory computer science courses, digital literacy modules, or even as part of humanities and social sciences classes, where students are taught to assess the reliability of AI-generated content. This interdisciplinary approach to AI literacy ensures that students from all academic backgrounds are prepared to use tools such as ChatGPT, regardless of their field of study. Tailoring AI tools, such as ChatGPT, to the specific needs of the educational community can further enhance their perceived usefulness. One way to do this is by gathering feedback from AI-literate students, who can provide detailed insights into the tool’s strengths and weaknesses in academic contexts. AI-literate users are likely to have a more nuanced understanding of what works well and what could be improved, enabling technology providers to make informed adjustments that meet the precise requirements of students and educators.

### 5.7. AI Literacy and Perceived Enjoyment

The support for H7 indicates that a higher level of AI literacy, or an understanding of AI concepts and capabilities, enhances students’ perceived enjoyment of using ChatGPT. This finding highlights the importance of not just the functional benefits of technology, but also how knowledge and comfort with the underlying technology can affect user experience. This is evident in the literature on digital literacy [64]. This study found that when users better grasp AI’s capabilities and limitations, they can engage with ChatGPT more effectively and creatively. This deeper engagement can lead to a more enjoyable experience, as users will explore a wider range of features and use cases, discovering novel and interesting ways to incorporate the tool into their academic and personal lives. When users understand what ChatGPT can and cannot do, they are more likely to use it in ways that yield successful outcomes, thereby enhancing the overall enjoyment of the experience. The positive impact of AI literacy on perceived enjoyment suggests a valuable role for AI education initiatives.

Educational institutions and technology providers can play a pivotal role in enhancing students’ engagement with AI tools like ChatGPT by offering a variety of programmes and resources. For instance, workshops and courses could provide students with a hands-on learning experience in which they are guided through practical applications of ChatGPT. These could include sessions on how to use ChatGPT for research, content generation, or problem solving. By focusing on practical, real-world applications, these workshops could also show students how to use the tool in academic and non-academic contexts, thus broadening their understanding of its capabilities. Courses could include step-by-step tutorials, guiding students through integrating ChatGPT into their daily academic tasks, from generating study guides to conducting literature reviews. These offerings would also emphasise best practices, helping students avoid common pitfalls, such as overreliance on the tool or failure to critically assess its output. In addition to formal instruction, creating online resources such, as video tutorials, articles, and how-to guides, could allow students to learn at their own pace. These resources should cover a range of topics, from basic introductions to AI concepts to more advanced strategies for using ChatGPT in specific academic tasks.

Highlighting the creative and unexpected uses of ChatGPT can also serve as a source of inspiration for students. Educational institutions and technology providers can curate and share case studies or student testimonials that showcase how ChatGPT has been used in innovative academic projects. For instance, a student might have used ChatGPT to develop a unique algorithm for data analysis or to generate a draft for a group project. Sharing these success stories in newsletters, online platforms, or even during classroom discussions can spark curiosity among students and encourage them to think outside the box. Additionally, highlighting how ChatGPT can be applied to personal development, such as learning new skills, setting goals, or exploring new areas of interest, can demonstrate its versatility. For example, ChatGPT could assist students with brainstorming ideas for personal projects, learning new languages, or organising their study schedules. Showcasing these non-academic uses can make the tool more relatable and enjoyable for students, making it not just a productivity tool, but a personal assistant that enhances multiple aspects of their lives.

### 5.8. AI Literacy and Perceived Risk

Given that H8 was not supported, perceived AI literacy did not positively influence the perceived risk of using ChatGPT. This finding offers a unique perspective on the correlation between students’ knowledge of AI technologies and their evaluation of the potential risks linked to these technologies—contrary to many studies that have proven a strong relationship between AI literacy and students’ perceived risk [66,67,68]. The lack of support for H8 suggests that a student’s understanding or knowledge of AI does not necessarily make them perceive ChatGPT as riskier. This could mean that AI-literate students feel confident in managing potential risks or that concerns about risk are more influenced by other factors, such as media reports, peer opinions or personal experiences, rather than AI literacy. An alternative interpretation could be that higher AI literacy leads to overconfidence in one’s ability to use ChatGPT without encountering risks. This overconfidence could mask the perception of risk, rather than demonstrate an actual reduction in risk, among more AI-literate users.

Given this, it is essential for educational institutions, developers, and policymakers to prioritise comprehensive education on the potential risks of AI tools, ensuring that these efforts reach all users regardless of their proficiency level. AI literacy programmes should emphasise the ethical, security, and practical risks of using tools such as ChatGPT. For example, users should be educated on issues such as data privacy, misinformation, bias, and reliance on AI for decision making, which can pose significant challenges if not effectively managed. This would help prevent AI-literate users from becoming overly reliant on the technology without critically assessing its limitations. To make risk awareness accessible, developers and educators should create content that is both relatable and easy to understand, regardless of a user’s AI background. Such content could include developing user-friendly guides, video tutorials or interactive modules that explain risks in a practical context. For instance, short videos could break down real-world scenarios in which AI tools might generate incorrect or biased outputs, teaching users how to identify and mitigate these risks. The key is to provide practical examples and actionable steps that users can take to avoid potential pitfalls, such as double-checking AI-generated content, ensuring data privacy, or understanding the tool’s limitations.

Institutions could also develop risk awareness workshops or seminars specifically focused on teaching safe and responsible usage of AI tools. These could address common misconceptions, such as the belief that more advanced users are inherently immune to AI’s risks. Discussions in these workshops might also explore AI’s societal and ethical implications, highlighting how tools like ChatGPT can inadvertently perpetuate bias or misinformation and offering strategies for users to mitigate these risks in their work. Continued research into the perceptions of risk associated with AI tools is also crucial. Understanding how different user groups—whether AI-literate or not—perceive and react to potential risks can help developers and educators tailor risk communication strategies. This could involve regularly engaging with users to gather feedback on their experiences, concerns and challenges with AI tools. Surveys, focus groups and user interviews could provide valuable insights into how users assess risk and whether current educational efforts around safe usage are effective.

### 5.9. AI Literacy and Perceived Fees

The support for H9 suggests that a higher level of AI literacy positively influences how students perceive the fees associated with ChatGPT. This finding is particularly insightful, as it highlights how users’ knowledge about AI can shape their awareness of AI technology costs [69]. Users with a higher degree of AI literacy may better understand the complexities involved in developing and maintaining AI technologies, such as ChatGPT. This understanding can lead to a greater appreciation of these technologies’ value, making users more amenable to paying for access to advanced features or services.

For educational providers offering ChatGPT, transparent communication about pricing is key to building trust and ensuring that users understand the value they receive in return for the fees they pay. Pricing models can sometimes seem arbitrary to users, especially with AI tools such as ChatGPT, where the technology behind the service might not be immediately apparent. Therefore, providers should focus on explaining the underlying technology, development efforts, and benefits associated with premium features to help users appreciate the costs involved. For example, educational providers can highlight the research and development that goes into creating and maintaining a tool like ChatGPT, including the complex algorithms, data processing capabilities, and continuous updates required to keep the system accurate and relevant. They could also emphasise the value-added features offered in premium versions—such as faster response times, enhanced capabilities, more personalised experiences or higher usage limits—that justify the pricing structure.

Additionally, introducing pay-per-use models or discounts for academic institutions can make ChatGPT more accessible to students and educators with varying budgets. Educational providers can also consider offering free trials or demo versions that allow users to experience premium features firsthand before committing to a paid subscription. This trial period can help users better understand how the advanced functionalities of ChatGPT can enhance their learning or productivity, making them more likely to perceive the fees as justified. Educational resources that improve users’ understanding of AI and ChatGPT can also be tied to the pricing model. For example, providers might offer bundled packages that include access to the tool and AI literacy courses or tutorials that teach users how to maximise their use of ChatGPT. This dual approach not only enhances the tool’s value but also helps users see the fees as an investment in their own learning and skill development.

### 5.10. AI Literacy and Perceived Value

The support for H10, which states that AI literacy positively influences the perceived value of ChatGPT, underscores the significance of students’ understanding of and familiarity with AI in appreciating the tool’s potential and contributions to their academic and personal endeavours. This was confirmed by other studies [22,52,58]. This relationship suggests that a deeper comprehension of AI not only enhances the usability of ChatGPT, but also elevates its overall importance and value in the eyes of the students. AI-literate students are more likely to understand the complexities and capabilities of ChatGPT, leading to an informed appreciation of its functionality. With a solid grasp of AI principles, students can strategize their use of ChatGPT more effectively, applying it to tasks that offer the most benefit.

The link between AI literacy and the perceived value of ChatGPT suggests a critical role for educational initiatives aimed at increasing AI literacy among students in higher education. Educational institutions might consider integrating AI literacy into their curricula or offering standalone courses and workshops focused on understanding AI literacy components. These could cover the components of AI literacy, including the ethical, social and technical aspects of AI, while also addressing practical usage. Workshops can be tailored to different user levels, from beginners who need an introduction to AI concepts to advanced students who want to explore AI’s more sophisticated applications in their research or projects. These workshops could provide hands-on practice with tools like ChatGPT, demonstrating how AI can assist in automating tasks, generating insights or improving learning outcomes. To further enhance the perceived value of ChatGPT, developers and educators can design guided learning experiences that showcase its full range of capabilities while teaching students how to use the tool effectively. For instance, interactive tutorials could walk students through specific tasks, such as using ChatGPT for brainstorming, writing assistance, or research summaries. These tutorials could be customised to reflect different academic disciplines, helping students see how ChatGPT can address their unique needs, whether they are writing a research paper in the literature or analysing data in engineering.

Challenge-based learning could also be employed to make the learning experience more engaging. In a challenge-based format, students are given real-world problems or projects where they are encouraged to use ChatGPT to generate solutions, analyse information or create content. For example, a class could conduct a mock research study using ChatGPT to generate hypotheses, summarise articles or identify gaps in the literature. This approach could increase students’ familiarity with the tool and encourage critical thinking about its capabilities and limitations. Such experiences help students move beyond passive use, promoting active engagement and problem solving.

Incorporating project work into the curriculum, where students must use AI tools like ChatGPT, could further solidify their understanding of the tool’s practical applications. Projects could range from individual assignments—where students use ChatGPT to draft essays or reports—to group projects, where students collaborate on larger tasks, such as developing presentations or conducting data-driven research. These projects could be evaluated on the final output and on students’ ability to ethically navigate AI tools, assessing how they balance reliance on AI with critical judgement. By fostering a culture of inquiry and ethical engagement, educational providers can help students recognise the practical value of ChatGPT and its broader implications in the academic world. Providing ongoing feedback loops with educators and students is critical for developers. Regular updates and improvements based on user experiences, including ethical concerns and practical challenges, will ensure that ChatGPT evolves to better meet the needs of the educational community.

## 6. Conclusions, Limitations, and Future Work

The current study seeks to verify students’ intentions to use ChatGPT in education and to highlight the perceived value of this tool and the influencing role of AI literacy in shaping these perceptions. This research is among the pioneering efforts to evaluate ChatGPT acceptance in higher education through an extended VAM framework with AI literacy. This factor has emerged in the AI era. Using this framework, educators and developers can more effectively customise ChatGPT to address the diverse needs of students, improving the adoption and effectiveness of its integration into learning. The findings suggest that perceived usefulness, enjoyment, and fees significantly affected students’ perceived value of using ChatGPT in learning, and perceived value had a significant role in driving students’ intention to use ChatGPT.

However, perceived risks had no significant influence on students’ perceived value. The results indicate that students’ AI literacy significantly and positively influences students’ perceived usefulness, enjoyment, fees, and value of ChatGPT and had no influence on students’ perceived risks. Investigating students’ use intentions through the combined lens of perceived value and AI literacy provides a nuanced understanding of the factors driving the adoption and effective use of ChatGPT in educational settings. It can also guide educators and policymakers in designing curricula and policies that integrate AI technologies, such as ChatGPT, seamlessly and prepare students to navigate the evolving AI landscape ethically and effectively.

Several potential limitations could impact the study’s outcomes and generalizability. Initially, this study relied on a convenience sampling method, selecting participants exclusively from several universities within the authors’ eastern region, limiting the findings’ applicability to the broader student population across Saudi Arabia. Furthermore, the data collection relied on electronic surveys distributed through university emails and social media, potentially including respondents unfamiliar or inexperienced in ChatGPT, which might have affected the study’s conclusions. In addition, focusing solely on VAM and AI literacy may overlook other critical factors influencing the acceptance and use of AI technologies.

Another limitation of this study is the treatment of AI literacy as a single unified construct. While this approach enabled us to assess its overall impact on other variables, it did not account for the individual components of AI literacy, such as ethical understanding, functional skills, and critical evaluation of AI technologies. These distinct aspects may independently influence students’ acceptance and adoption of generative AI tools like ChatGPT. Addressing these limitations in future research could involve broadening the sampling strategy to include a wider range of universities and student demographics. Criteria could be established to include participants with experience in utilising ChatGPT for educational purposes. The research framework for considering additional factors and their interactions could be expanded.

Future research could expand the model to include environmental factors, such as institutional support, available resources and the integration of ChatGPT into the curriculum. Likewise, future studies could investigate psychological factors, including trust in AI and perceived control over the technology. Furthermore, future studies could consider the moderating effects of demographic variables, such as age, gender, academic major and prior experience with AI technologies. Future research should also investigate AI literacy components to better understand how each component shapes students’ perceptions, behaviours, and engagement with AI in educational contexts.

## Figures and Tables

**Table 1:** Sample outline (*n* = 676).

Features	*n*	%
**Gender**		
Male	109	16.1
Female	567	83.9
**Age**		
≤18	103	15.2
19–20	294	43.5
21–22	170	25.1
23–24	41	6.1
≥25	68	10.1
**Education Level**		
Undergraduate	619	91.6
Graduate	57	8.4
**Academic Major**		
Health sciences	118	17.5
Agriculture	193	28.6
Humanities	44	6.5
Social sciences	23	3.4
Pure sciences	60	8.9
Computer science	238	35.2

**Table 2:** Analysis of the measurement model.

Construct	Indicator (In)	Standardized Indicator Loadings	α	CR	AVE	R^2^	R^2^ Adjusted	Q^2^
Perceived Usefulness	In 1	0.91	0.892	0.892	0.822	0.263	0.262	0.259
	In 2	0.90						
	In 3	0.89						
Perceived Enjoyment	In 1	0.91	0.896	0.896	0.827	0.292	0.291	0.289
	In 2	0.92						
	In 3	0.89						
Perceived Risk	In 1	0.89	0.857	0.971	0.601	0.019	0.017	0.011
	In 2	0.76						
	In 3	0.84						
	In 4	0.68						
	In 5	0.72						
Perceived Fees	In 1	0.89	0.802	0.803	0.835	0.045	0.052	0.049
	In 2	0.91						
	In 3	0.92						
Perceived Value	In 1	0.72	0.862	0.889	0.712	0.624	0.621	0.282
	In 2	0.90						
	In 3	0.90						
	In 4	0.87						
Use Intention	In 1	0.90	0.914	0.918	0.796	0.560	0.560	0.254
	In 2	0.91						
	In 3	0.93						
	In 4	0.82						
AI Literacy	In 1	0.75	0.929	0.936	0.611			
	In 2	0.80						
	In 3	0.82						
	In 4	0.81						
	In 5	0.77						
	In 6	0.81						
	In 7	0.83						
	In 8	0.77						
	In 9	0.75						
	In 10	0.72						

**Table 3:** Analysis of discriminant validity.

Constructs	1	2	3	4	5	6	7
1. Perceived Usefulness	**0.906**						
2. Perceived Enjoyment	0.761 (0.801)	**0.909**					
3. Perceived Risk	−0.095 (0.072)	−0.079 (0.082)	**0.774**				
4. Perceived Fees	0.274 (0.324)	0.313 (0.368)	−0.044 (0.091)	**0.912**			
5. Perceived Value	0.729 (0.813)	0.693 (0.771)	−0.030 (0.081)	0.397 (0.495)	**0.844**		
6. Use Intention	0.653 (0.721)	0.583 (0.642)	−0.032 (0.075)	0.233 (0.270)	0.741 (0.831)	**0.898**	
7. AI Literacy	0.513 (0.553)	0.541 (0.538)	−0.137 (0.120)	0.232 (0.263)	0.535 (0.582)	0.520 (0.554)	**0.782**

The bold figures denote the square roots of the AVE, with values in parentheses representing the HTMT ratios.

**Table 4:** Hypothesis testing result.

H	Independent Variables	Path	Dependent Variables	β	SE	t	*p*
H1	Perceived Usefulness	→	Perceived Value	0.433	0.049	8.830	0.000 *
H2	Perceived Enjoyment	→	Perceived Value	0.226	0.048	4.727	0.000 *
H3	Perceived Risk	→	Perceived Value	0.054	0.050	1.172	0.241
H4	Perceived Fees	→	Perceived Value	0.172	0.030	5.801	0.000 *
H5	Perceived Value	→	Use Intention	0.748	0.022	33.70	0.000 *
H6	AI Literacy	→	Perceived Usefulness	0.513	0.035	14.54	0.000 *
H7	AI Literacy	→	Perceived Enjoyment	0.541	0.032	18.82	0.000 *
H8	AI Literacy	→	Perceived Risk	−0.137	0.093	1.473	0.141
H9	AI Literacy	→	Perceived Fees	0.232	0.043	5.429	0.000 *
H10	AI Literacy	→	Perceived Value	0.158	0.34	4.600	0.000 *

* Significant at *p*-value < 0.001.

## Data Availability

The data is not available due to confidentiality concerns.
